# Structure Zone Investigation of Multiple Principle Element Alloy Thin Films as Optimization for Nanoindentation Measurements

**DOI:** 10.3390/ma13092113

**Published:** 2020-05-02

**Authors:** Alan Savan, Timo Allermann, Xiao Wang, Dario Grochla, Lars Banko, Yordan Kalchev, Aleksander Kostka, Janine Pfetzing-Micklich, Alfred Ludwig

**Affiliations:** 1 Materials Discovery and Interfaces, Institute for Materials, Ruhr University Bochum, 44780 Bochum, Germany; alan.savan@rub.de (A.S.); timo.allermann@rub.de (T.A.); xiao.wang@rub.de (X.W.); dario.grochla@rub.de (D.G.); lars.banko@rub.de (L.B.); 2 Werkstoffwissenschaft, Institut für Werkstoffe, Fakultät Maschinenbau, Ruhr University Bochum, 44780 Bochum, Germany; yordan.kalchev@rub.de; 3 Zentrum für Grenzflächendominierte Höchstleistungswerkstoffe (ZGH), Ruhr University Bochum, 44780 Bochum, Germany; aleksander.kostka@rub.de (A.K.); janine.pfetzing@rub.de (J.P.-M.)

**Keywords:** thin films, microstructure, high entropy alloys, complex solid solution, multiple principal element alloys, sputtering

## Abstract

Multiple principal element alloys, also often referred to as compositionally complex alloys or high entropy alloys, present extreme challenges to characterize. They show a vast, multidimensional composition space that merits detailed investigation and optimization to identify compositions and to map the composition ranges where useful properties are maintained. Combinatorial thin film material libraries are a cost-effective and efficient way to create directly comparable, controlled composition variations. Characterizing them comes with its own challenges, including the need for high-speed, automated measurements of dozens to hundreds or more compositions to be screened. By selecting an appropriate thin film morphology through predictable control of critical deposition parameters, representative measured values can be obtained with less scatter, i.e., requiring fewer measurement repetitions for each particular composition. In the present study, equiatomic CoCrFeNi was grown by magnetron sputtering in different locations in the structure zone diagram applied to multinary element alloys, followed by microstructural and morphological characterizations. Increasing the energy input to the deposition process by increased temperature and adding high-power impulse magnetron sputtering (HiPIMS) plasma generators led to denser, more homogeneous morphologies with smoother surfaces until recrystallization and grain boundary grooving began. Growth at 300 °C, even without the extra particle energy input of HiPIMS generators, led to consistently repeatable nanoindentation load–displacement curves and the resulting hardness and Young’s modulus values.

## 1. Introduction

High entropy alloys (HEAs), also referred to as multiple principal element alloys (MPEAs) or compositionally complex alloys (CCAs), are multinary materials where the constituent elements are in, or near, equiatomic concentrations. This is in contrast to traditionally developed alloys, which are based on one or two elements as the principal components with small amounts of additional elements that add, enhance, or adjust certain properties. Although experience from conventional metallurgy has led to the expectation that MPEAs will be multiphase and will usually have intermetallic or other complicated compounds and microstructures resulting in poor mechanical properties, some MPEAs have been discovered that form single-phase solid solutions with a simple fcc or bcc crystal structure [1,2,3,4].

The composition-based definition of high entropy materials is the presence of five elements or more, each with an atomic percentage between 5% and 35% [2]. A configurational entropy-based definition would lead to materials with a mixing entropy equal to or greater than 1.5R (where R is the gas constant, 8.314 J·K^−1^ mol^−1^) and a system where five or more elements would have a greater probability of forming solid solutions [5]. However, Otto et al. have shown that high configurational entropy is not a determinant in countering the driving forces leading to secondary phases and that the criteria of using five or more equiatomic elements for anticipating a single-phase solid solution is insufficient [6]. It is also worth noting that current high-temperature materials, such as Ni-based superalloys, depend on having a second phase to achieve their outstanding properties. Then, the investigation of MPEA systems extends beyond the search for single-phase solid solutions.

Thus, just in terms of the number of possible unique combinations of five elements using only 30 solid, commercially available, relatively nontoxic, metallic elements, there are over 140,000 possibilities. Each of those systems then has a five-dimensional composition space as each of the components is varied away from equiatomic. Conventional metallurgical investigations are used for single-composition bulk samples, which are then exhaustively studied to determine and understand their structure and properties. Because of the intense requirements of time, energy, and material use required to produce these samples, studies typically cluster around compositions with a high proportion of one or two elements or the multielement equiatomic composition, where previous experience indicates a high-enough probability of practical importance to justify the expenses. The search through high-dimensional composition space to determine the existence range for properties found at a particular composition, to map multidimensional trends for property optimization, and to search for unexpected microstructures and properties or combinations of properties leads to the need for higher speed and greater efficiencies in the use of laboratory resources.

Combinatorial material synthesis and high-speed automated characterization, also referred to as “high-throughput characterization”, is an effective method to search for promising MPEA compositions [7,8] and also for investigating the concentration ranges around already-identified MPEAs where a single-phase solid solution or particular properties of interest are maintained. It is also beneficial in testing theoretical predictions, where the use of estimations for constants and factors in the model equations leads to probable imprecision in the calculated results. The use of thin films enables both a rapid means of creating combinatorial material libraries and efficient use of material. However, this method also brings challenges stemming from the characteristics of thin films and the necessarily automated, high-speed measurements to characterize the dozens, hundreds, or more individual compositions within combinatorial libraries. Even so, such high-throughput characterization of thin film libraries has been demonstrated to successfully identify regions of interest, which can subsequently be used to guide the making of bulk material with that composition showing the same phases and properties [9,10,11]. The thin film combinatorial approach is a systematic technique to identify candidates for detailed, comprehensive bulk material and thin film material study of alloys of scientific interest and/or practical importance, and it is therefore an addition and complement to the traditional approach of one alloy composition at a time investigation.

MPEAs have been produced by many different methods, such as arc melting, mechanical alloying, and spark sintering. When grown by physical vapor deposition (PVD), high-purity material can be made in thin film form with fine control of composition and directly grown atomic-scale mixing of the elements. The deposition parameters used during this process affect the microstructure of the film, which then influences the extrinsic properties of the material.

PVD-grown films with thickness in the range of 1 µm and greater develop morphologies that can be categorized by structure zone diagrams (SZD) [12,13]. In SZD, the film morphologies are shown as a function of the dominant growth parameter(s) that lead them to develop. The first of these to be observed is the homologous temperature, T/T_melt_, but other sources of energy to the condensing particles can also be significant. In this study, we used temperature and particle energy resulting from the input power to the plasma used for the sputtering process [14] to obtain films in different structural zones.

In particular, high-power impulse magnetron sputtering (HiPIMS) produces a flux of particles that contains a significantly higher fraction of singly and doubly charged metal ions that arrive at the growth surface [15] compared to what is produced when direct current (DC) or radio frequency (RF, 13.56 MHz) generators are used instead. Among other effects, this additional energy can modify the structure of the condensed film [16]. As a practical constraint, we had available up to two DC, three RF, and two HiPIMS generators, each of which could be connected to any one of up to five sputter cathodes. Therefore, we compared films grown with a set of two DC and two RF generators to films grown with one DC, one RF, and two HiPIMS generators.

Equiatomic CoCrFeMnNi was one of the initial MPEAs to be widely investigated [1] because of its technologically important, relatively earth-abundant constituents and because it has been observed to form a single-phase solid solution when it is in (or near) equiatomic concentration. Furthermore, several of the unary, binary, and ternary alloys from these elements have important properties and are in widespread use. Among the quaternary or “medium entropy alloy” systems, CoCrFeNi is of interest because it can form a single-phase fcc solid solution with phase stability to at least medium temperatures [17,18]. While CoCrFeNi has interesting deformation behavior at high strain [19,20], it was selected here mainly because it is a MPEA with a widely applicable basis for achieving tailored properties by adjusting the concentrations of the constituent elements [21] or by adding others, such as in Mn, Al, Cu, Ti, etc. [22,23,24,25].

The growth of dense and smooth thin films of multinary materials is required for determining their mechanical properties using nanoindentation [26], especially for high-throughput nanoindentation approaches. For nanoindentation, a film thickness of 1 µm or higher is recommended so that the mechanical properties of the substrate are not confounded with those of the film. However, higher thin film thickness often also increases the surface roughness. If the films are too rough, the measured values of Young’s modulus and hardness tend to scatter. This problem is especially significant for the investigation of thin film combinatorial material libraries, where the effects of intentional composition gradients are being characterized and a wide spread in measurement values at specific composition points can obscure composition-to-composition trends or abrupt changes, e.g., if there is a phase region boundary. Another issue that can arise is the local formation of voids, pores, or gaps between adjacent grains and cracks forming perpendicular to the substrate surface, which also affect the apparent nanoindentation measurement value at or near that feature and result in a misleading data point. As a practical matter, when measuring dozens to hundreds of unique composition locations as may be necessary when concentrations of four or more elements are simultaneously varying across a combinatorial material library, only a limited number of nanoindentation repetitions, for instance, 3–5, can be made at each location. Identifying and handling data points that might be outliers becomes time-consuming and difficult, and it is effectively impossible to reliably distinguish which ones are actually measurement failures.

Here, we investigated how to better achieve MPEA thin film microstructures that are suitable for high-throughput mechanical property screening using Co–Cr–Fe–Ni as a basis model system and applying different magnetron sputter growth process conditions.

## 2. Materials and Methods

The thin films were magnetron sputtered using the confocal codeposition chamber of a combinatorial system (600/400 LIN UHV deposition system, DCA Finland) [27]. Sapphire (c-plane) was used as substrates due to its excellent chemical stability and because the materials being deposited adhere well to it at all the tested temperatures. The substrates were kept at a constant temperature established prior to deposition: 25, 300, 500, and 900 °C. A backside thermocouple within the substrate heater was previously calibrated to these substrate temperatures using a separate test substrate with embedded frontside thermocouples for temperatures up to 425 °C and an infrared pyrometer for higher temperatures. Each element was sputtered from individual Ø100 mm targets: Cr (99.95% MaTecK GmbH, Jülich, Germany), Co (99.99% MaTecK), Fe (99.99% Evochem GmbH, Offenbach, Germany), and Ni (99.995% K.J. Lesker Co., Hastings, UK). All targets were precleaned by sputtering against closed, individual-target shutters immediately prior to thin film growth. The power supplies applied to the individual, elemental targets were (1) direct current to the Cr and Fe targets and radio frequency (13.56 MHz) to the Co and Ni targets and (2) DC to Fe, RF to Ni, and HiPIMS to Cr and Co. The available generators were two DC (5 kW RPG-50, ENI USA) and three RF (600 W, 13.56 MHz, AGC-6B ENI). Being a strongly magnetic material, the Fe target was constrained to be DC. The remaining targets were assigned to power supplies such that their deposition rates could be matched to that of Fe with well-controllable power ranges. There were an additional two HiPIMS (6 kW GX 1000V Dual with SIPP-10-1000-S pulse controller, MELEC, Baden-Baden, Germany). Here, Cr and Co were selected based on their ionization characteristics in HiPIMS discharges, facilitating control of peak current and metal/gas ion ratio with voltage, current. and pulsing parameters [15,28,29,30]. The power to each target was adjusted such that composition close to equiatomic was achieved for all films sputtered using argon (purity 99.9999%) at a pressure of 0.667 Pa. A substrate rotation rate of 10 rpm was used to ensure compositional uniformity. The total deposition rate was determined for each experimental condition, and then each film was grown to have a comparable thickness of approximately 1.2 µm. The film thickness was determined by stylus profilometry of photolithographically patterned substrates deposited at room temperature after adjusting the elemental composition to be equiatomic. Actual film thickness for films deposited at the various temperatures were determined by scanning electron microscopy (SEM) cross sections. The growth rate of the quaternary element films was approximately 0.14 nm/s.

The elemental composition of the films was characterized by energy-dispersive X-ray spectroscopy (EDS, Inca X-act, Oxford Instruments) at 20 keV using a Co calibration standard, yielding element concentrations with an accuracy of about 1 atomic percent. X-ray diffraction (XRD) patterns were measured with a Bruker D8 Discover using filtered Cu Kα radiation in the 2-Θ range of 30–110°.

SEM was performed with a JEOL JSM-7200F, with further image analysis then performed using ImageJ (Fiji) software. Additional surface characterization was made with a Keyence VK-X200 series confocal laser scanning microscope (CLSM). Transmission electron microscopy (TEM) was done in a Tecnai Supertwin F20 G2 operating at 200 kV. TEM samples were prepared by focused ion beam (FIB) milling in a FEI Helios G4 CX dual beam system using a Ga ion beam.

Atomic force microscopy (AFM) characterization of surface topography was made with a Bruker Dimension instrument running in tapping mode. Hardness and Young’s modulus were measured with a Nano Indenter XP (MTS Nano Instruments) with a Berkovich indenter tip calibrated on fused silica using the Oliver–Pharr method [31] operated in a Standard CSM (continuous stiffness measurement) test mode. Maximum indentation depth was 100 nm, and 10 indentations, spaced 15 µm apart, were made for each thin film. These were later located and imaged by SEM. Mean values for modulus and hardness and the standard deviations were calculated for each condition.

## 3. Results and Discussion

Eight experiments with four different temperatures and two sets of power supply configurations were conducted (Table 1). The deposition times were adjusted to yield about 1.2 µm thickness for each condition. The homologous temperature, T_m_, of equiatomic CoCrFeNi was estimated using the rule of mixtures to be 1870 K. The selected temperatures corresponded to SZD zones 1, T, and 2, depending on the axes of the particular SZD model being used. It should be noted that the temperatures referred to are the calibrated substrate temperatures established by the substrate heater. They do not account for the local surface temperature of the growing film coming from the energy of arriving particles [14].

### 3.1. Crystal Structure, Phase Constitution

**Figure 1:**
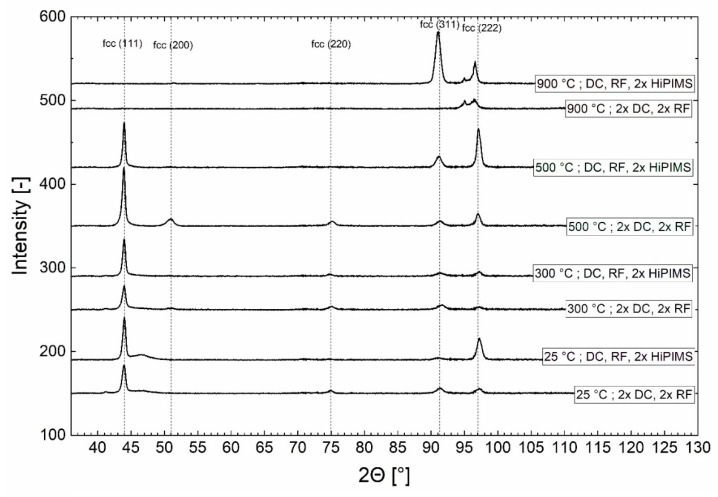
Results of XRD for eight as-deposited CrCoFeNi films deposited at substrate temperatures of 25, 300, 600, and 900 °C with two different power supply arrangements, indicating fcc crystal structure.

The results of the XRD analysis are shown in Figure 1. For all films, except the ones deposited at 900 °C, the dominant peak corresponded to the (111) plane of the fcc structure. Further peaks from the fcc phase were identified as (200), (220), (311), and (222) planes [32,33,34]. Films grown with both power supply arrangements at 900 °C had an additional peak appearing at 95°.

The peak at 95° did not fit to an fcc phase, but it was alone not sufficient to uniquely identify its source in such a highly textured thin film. Possible candidates with an XRD peak in this location are Mn, CoMn, MnNi, sigma phase, CoCr, and CrFe. As the 900 °C film morphology was indicated to be unsuitable for high-throughput nanoindentation screening (see Section 3.4, below), further, more specific phase analysis was not conducted as part of this study.

### 3.2. Surface Morphology

**Figure 2:**
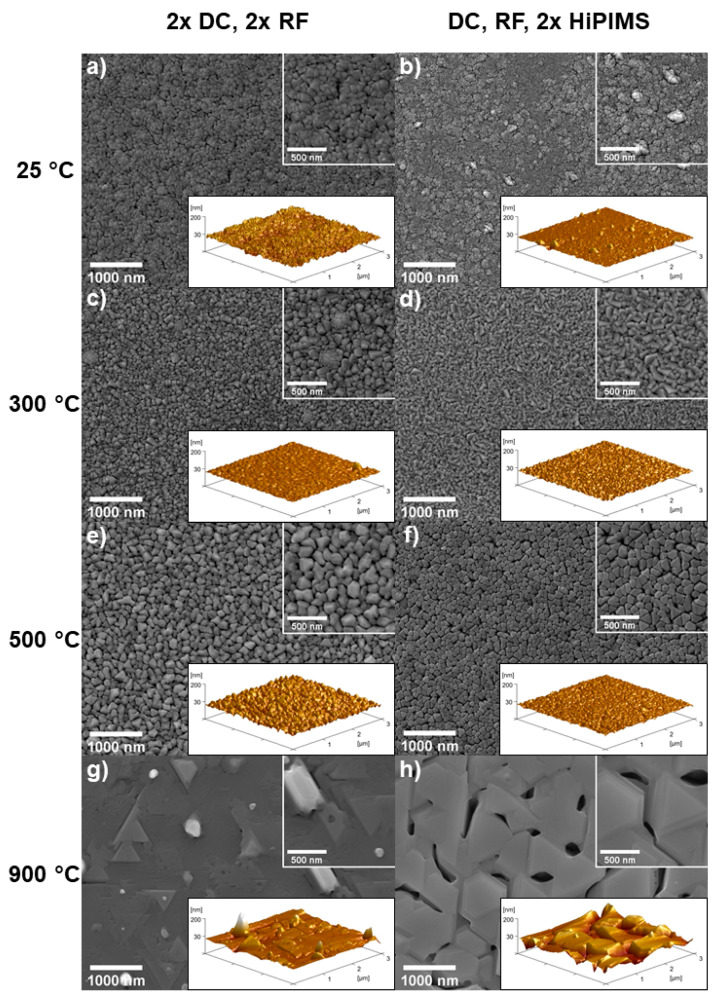
SEM and atomic force microscopy (AFM; inset) images of the surface topography of all CoCrFeNi films. Films shown in the left column (**a**,**c**,**e**,**g**) were grown with two cathodes powered by direct current (DC) and two cathodes powered by radiofrequency (RF) generators. The right column (**b**,**d**,**f**,**h**) films had cathodes powered by one DC, one RF, and two high-power impulse magnetron sputtering (HiPIMS) generators. Substrate temperatures during growth for each condition are indicated for each row.

Figure 2 gives an overview of the surface microstructure of all films. For room-temperature depositions, a fine-grained microstructure was observed. The films deposited at 25 °C (Figure 2a,b) had small grains, which were packed into bundles. Pores were noted at the surface of the 2 × DC, 2 × RF condition. Less uniform shapes and heights of the features, with scattered larger abnormalities, could be seen on the surface of the film with DC, RF, 2 × HiPIMS condition (Figure 2b). The surfaces had characteristics typical of structures obtained in both zone 1 and zone T in SZDs. The 2 × DC, 2 × RF film (Figure 2a) had a rougher surface than the DC, RF, 2 × HiPIMS condition (Figure 2b), even though the latter had larger abnormalities. AFM confirmed that these large abnormalities were protruding features.

In the 300 °C, 2 × DC, 2 × RF condition (Figure 2c), somewhat bigger clusters or bundles of grains could be seen. The surface was smoother, except at these clusters. One of them could be seen in the right corner of the AFM image, similar to those seen in Figure 2b. The 300 °C, DC, RF, 2 × HiPIMS sample (Figure 2d) had grains that became more facetted and had more elongated shapes, with the abnormal clusters largely suppressed.

At 500 °C (Figure 2e,f), both films had homogeneous surface features. The 2 × DC, 2 × RF condition (Figure 2e) was rougher than the films with lower deposition temperature in terms of peak-to-valley height differences. The DC, RF, 2 × HiPIMS condition (Figure 2f) seemed to have flatter grain tops but deeper pits at places where multiple grains meet. Some grains had triangular shapes.

Both 900 °C films were recrystallized. The 2 × DC, 2 × RF case (Figure 2g) had some triangular features and scattered protrusions with either round or rectangular cross sections. The DC, RF, 2 × HiPIMS film (Figure 2h) had much bigger grains compared to those formed at lower temperatures. They had triangular and hexagonal shapes with varying heights and indications of terraces and were partly fused together with large, deep voids in between the grains. Both of the 900 °C films had significantly different surface structures compared to all cases at lower temperature.

Thus, although there are four different elements, in the case of equiatomic (and near-equiatomic) CoCrFeNi, they grow as a single-phase solid solution until zone 3, and the resulting thin film morphologies follow the typical SZD with a rule of mixture estimation of the homologous temperature being adequate. This estimation yields a T/Tm of 0.63 for the experimental condition temperature of 900 °C during film growth, but the resulting morphology resembles the recrystallized grain structure of zone 3. With the further increase in energy from ionized species due to the HiPIMS plasma generators applied to two of the four elemental targets, there are features consistent with grain boundary grooving and the nucleation of dewetting holes.

The surface roughness parameter arithmetical mean deviation, R_a_, was determined by AFM (3 × 3 µm^2^ measurement area) and CLSM (12 × 9 µm^2^ measurement area) and are listed in Table 2. The 25 °C, DC, RF, 2 × HiPIMS condition (Figure 2b) had the smoothest surface (R_a_,_AFM_ = 3.0 nm, R_a_,_CLSM_ = 2.0 nm), even though some surface abnormalities were present. It was followed by the two 300 °C conditions with R_a_,_AFM_ = 3.5 (Figure 2c) and R_a_,_AFM_ = 3.3 nm (Figure 2d). The films deposited with HiPIMS at 900 °C showed the highest roughness. While the different measurement areas and resolutions of the AFM and CLSM led to different absolute values of R_a_, the trends were consistent within each set.

As some of the grains of the films deposited at 25 °C were grown together in bundles, it was hard to definitively distinguish each grain with an algorithm that could be consistently applied to each image. However, it might still be possible to produce useful estimates to quantify trends. In the cases of 300 and 500 °C, where more distinct grains were present, image processing algorithms could distinguish between grains, thus giving a significant count for the total number of grains and the grain size. For recrystallized microstructures, as observed for films grown at 900 °C, reliable values for grain counts and sizes could not be found with the same method. Consistent edge detection criteria could not properly distinguish between grains and surface features on grains and cut some of the big grains into several grains or counted terrace edges as separate grain boundaries. Thus, the 900 °C films were not included in further characterization (number of grains, grain size distribution, grain size).

The grain counts determined by image analysis from SEM micrographs at 20,000× magnification are shown in Table 3. The 25 °C, DC, RF, 2 × HiPIMS condition (Figure 2b) had the highest number of grains with 6200 counts. With 1436 counts, the 500 °C, 2 × DC, 2 × RF condition (Figure 2e) had the lowest number of grains. With increasing deposition temperature, the number of grains decreased. For each growth temperature, the corresponding film grown with DC, RF, 2 × HiPIMS had more grains than the ones grown with 2 × DC, 2 × RF.

**Figure 3:**
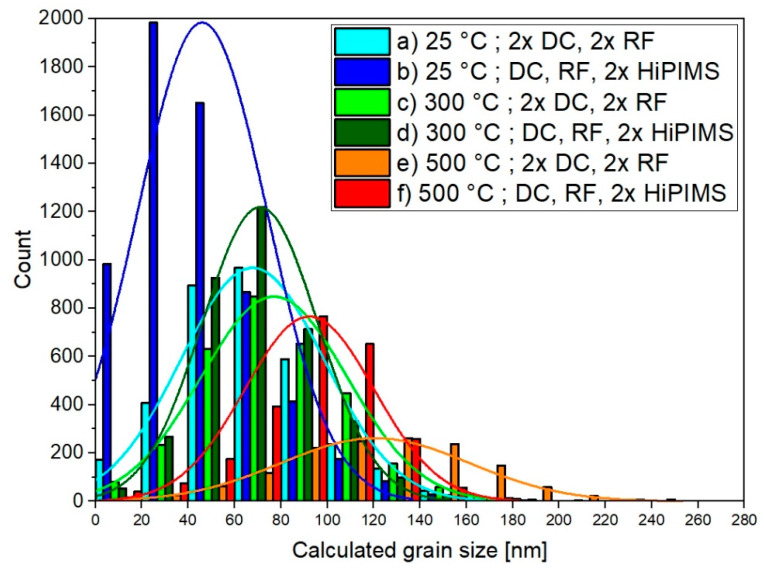
Calculated equivalent grain size distribution for each processing condition based on replacing the area of each grain at the surface extracted by image processing with an equivalent circular area. The lines are normal distributions fitted to each histogram as a guide for the eye.

Furthermore, the area of each grain (nm²) was determined from the processed images. Then, an equivalent grain size (nm) was calculated, assuming that each grain was circular. To give an overview about the grain size distribution, a histogram with a bin width of 20 nm is presented in Figure 3, with a normal distribution curve fitted to each histogram as a guide.

The DC, RF, 2 × HiPIMS conditions had a higher number of grains and smaller grains compared to the 2 × DC, 2 × RF conditions. However, for both cases, the grain size increased with increasing deposition temperature. The grain size distributions of the DC, RF, 2 × HiPIMS conditions were narrower than the grain size distributions of the corresponding 2 × DC, 2 × RF conditions.

### 3.3. TEM Analysis of Film Cross Sections

All films deposited at 25, 300, and 500 °C had a fine-grained crystallite nucleation region at the substrate interface. After a few tens of nanometers, faster-growing grains covered the slower ones, and a characteristic morphology developed.

**Figure 4:**
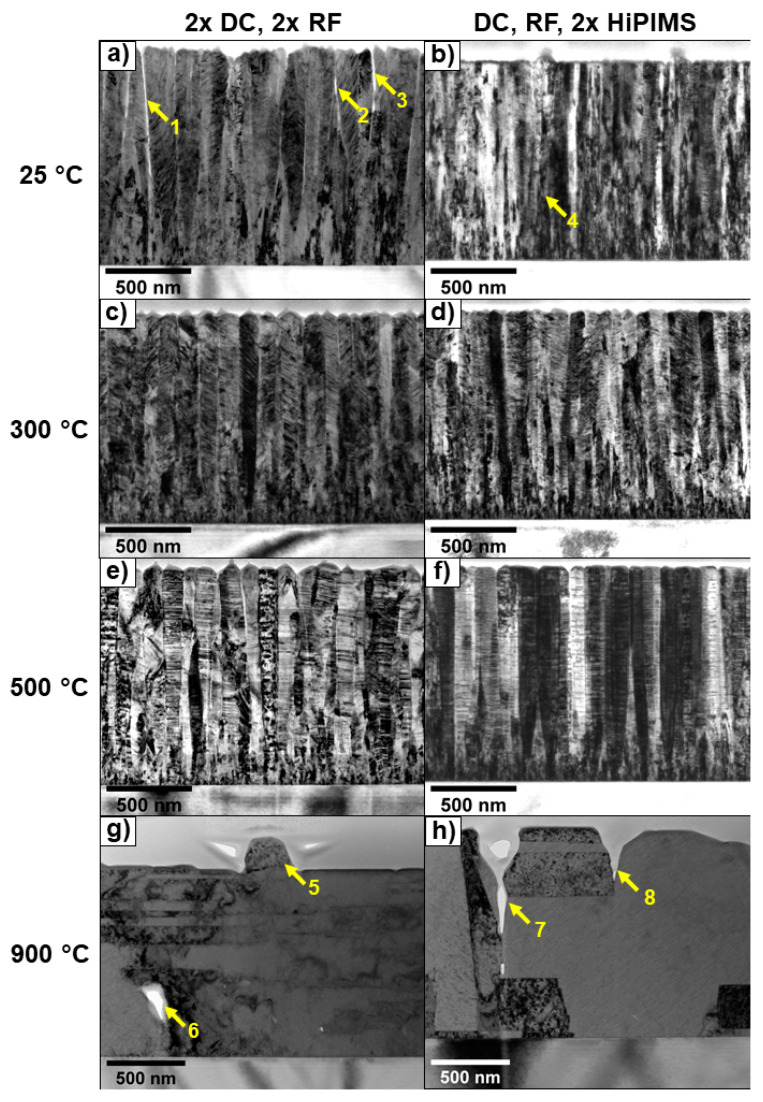
TEM images of focused ion beam (FIB) cross sections of CoCrFeNi films deposited at different process parameters. The left column films (**a**,**c**,**e**,**g**) were grown with two cathodes powered by DC and two cathodes powered by RF generators. The right column films (**b**,**d**,**f**,**h**) had cathodes powered by one DC, one RF, and two HiPIMS generators. Substrate temperatures during growth for each condition are indicated for each row. (**a**) Details 1, 2, and 3 highlight voids originating between grains. (**b**) Detail 4 indicates the origination of one of the abnormal surface features. (**g**) Detail 5 shows a large surface protrusion, and detail 6 shows an internal void. (**h**) Details 7 and 8 indicate intergranular grooves.

The 25 °C, 2 × DC, 2 × RF film (Figure 4a) had an inhomogeneous structure along the film thickness. V-shaped grains grew after burying the fine grains at the substrate interface. These V-shaped grains became grouped into bundles. Some grains were separated by voids (details 1, 2, and 3, Figure 4a). The 25 °C, DC, RF, 2 × HiPIMS film (Figure 4b) was also composed of small columnar grains that had collected into bundles. The “starting zone”, where slower-growing grains were buried, was smaller compared to the 25 °C, 2 × DC, 2 × RF film (Figure 4a), and the fast-growing grains tended to be narrower and had straighter boundaries, leading to a dense, void-free morphology. One of the larger surface abnormalities could be seen originating at about one-quarter of the ultimate film thickness (Figure 4b, detail 4). Both of the films deposited at 25 °C exhibited features characteristic of zone 1 and zone T in SZDs.

Both the 300 °C films (Figure 4c,d) were dense and had characteristics of zone T and zone 2. A small, fine-grained nucleation zone was present at the substrate interface. After that, the grains grew in rather straight, compact columns. The 2 × DC, 2 × RF film (Figure 4c) had a homogeneous structure along the film thickness. Some of the grains began as V-shaped, but most of the grain boundaries developed nearly perpendicular to the film plane. The grain boundaries of the 300° C, DC, RF, 2 × HiPIMS film (Figure 4d) were not as perpendicular to the film plane as in the case of the 300° C, 2 × DC, 2 × RF film (Figure 4c).

Similar to the films deposited at 300 °C, a small nucleation zone was present in the films deposited at 500 °C (Figure 4e,f). The developed grain size was bigger compared to lower-temperature films. Both films were homogeneous along the film thickness. Some of the grains started as V-shaped, but most of the grain boundaries were nearly perpendicular to the film plane. Both films appeared to be dense. These were characteristics of zone T and more particularly zone 2 in SZDs.

Both films deposited at 900 °C (Figure 4g,h) were recrystallized, and the grains were no longer columnar. In the 2 × DC, 2 × RF film (Figure 4g), a protruding feature could be seen in detail 5 extending approximately 200 nm out of the surface. The film was not dense; a void could be seen (Figure 4g, detail 6). In the DC, RF, 2 × HiPIMS film (Figure 4h), gaps between grains were present of about 670 nm deep (detail 7) and about 350 nm deep (detail 8). Despite these features, the films deposited at 900 °C had characteristics of zone 3 in SZDs.

The grain size (widths) determined in TEM images of FIB cross sections (Table 4) followed the same trends as the calculated equivalent mean circular grain sizes extracted from the image-processed SEM surface micrographs (Table 5).

### 3.4. Mechanical Properties

**Figure 5:**
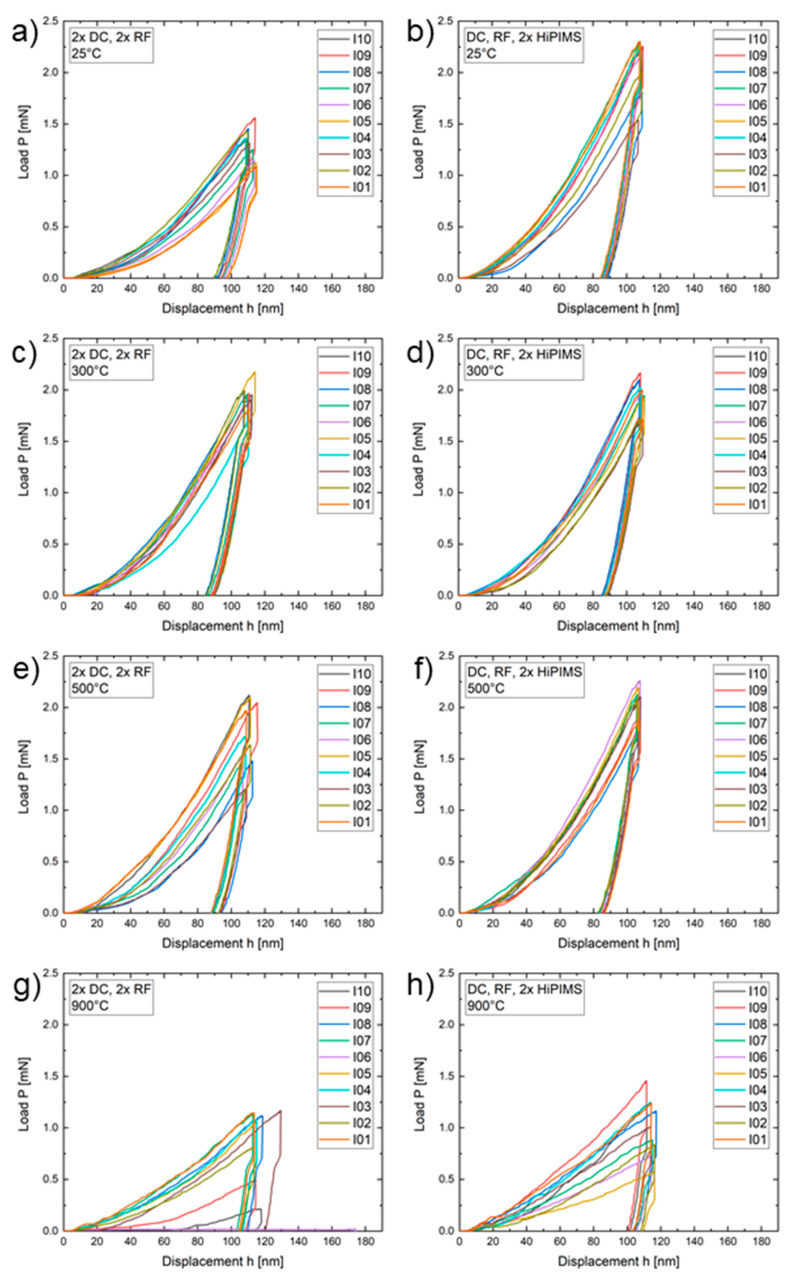
Nanoindentation load–displacement results of 10 indents spaced 15 µm apart with a final indentation depth of 100 nm. (**a**–**h**) The power supply arrangements and deposition temperature for each CrCoFeNi sample.

The nanoindentation load–displacement curves recorded for each experimental condition are shown in Figure 5. Note that the drop in maximum load for each curve is an artifact related to system calibration and is not a feature of the films.

Both of the 300 °C films (Figure 5c,d) with a maximum load (P_max_) in the range of 1.7–2.2 mN as well as the 500 °C, DC, RF, 2 × HiPIMS film (Figure 5f) with P_max_ in the range of 1.8–2.3 mN had the most consistently repeatable load–displacement curves. They were closely followed by the 25 °C, 2 × DC, 2 × RF condition (Figure 5a) with a maximum load in the range of 1–1.6 mN. The load–displacement curves of the 25 °C, DC, RF, 2 × HiPIMS film (Figure 5b) had a P_max_ in the range of 1.5–2.3 mN, while the 500 °C, 2 × DC, 2 × RF film (Figure 5e) with P_max_ in the range of 1.2–2.1 mN showed further increasing amounts of measurement scatter. The load–displacement curves of both 900 °C conditions (Figure 5g,h) had the most variability. The 900 °C, 2 × DC, 2 × RF condition had P_max_ in the range of 0.2–1.2 mN with one outlier of P_max_ = 0.02 mN. A maximum load in the range of 0.6–1.45 mN was determined for the 900 °C, DC, RF, 2 × HiPIMS condition.

**Figure 6:**
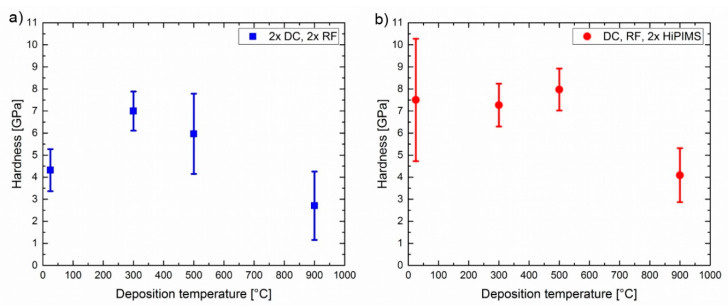
Hardness values as determined by nanoindentation of films deposited at different temperatures: (**a**) cathodes powered by two each DC and RF plasma generators. (**b**) cathodes powered by one DC and one RF generator, and two HiPIMS generators.

**Figure 7:**
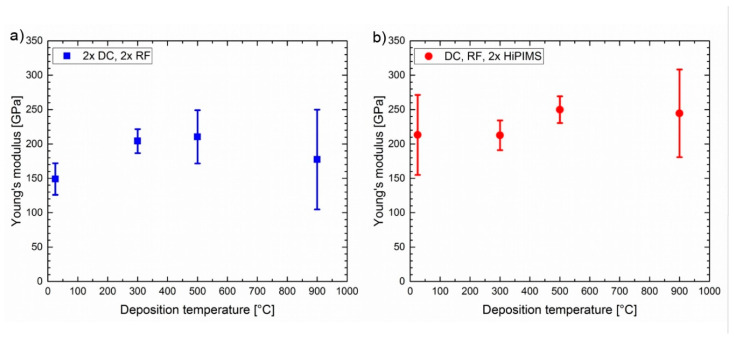
Young’s modulus values as determined by nanoindentation of films deposited at different temperatures, indicating the average, maximum, and minimum of 10 measurements: (**a**) cathodes powered by two DC and two RF plasma generators, (**b**) cathodes powered by one DC, one RF, and two HiPIMS generators.

Hardness and Young’s modulus calculated from the load–displacement curves are shown in Figure 6 and Figure 7, respectively. The error bars indicate the standard deviation of the values measured for 10 separate indents at each experimental condition. The hardness for the CoCrFeNi thin film varied from 2.9 (2 ×DC, 2 × RF, 900 °C) to 8 (DC, RF, 2 × HiPIMS, 500 °C) GPa. In general, higher energy inputs during deposition resulted in lower hardness values. This is probably due to recovery and recrystallization processes associated with larger grains (compare Figure 4g,h). In line with this, the Young’s modulus was less dependent on deposition parameters than the hardness measurements, with values from 150 (2 ×DC, 2 ×RF, 25 °C) to 240 (2 ×DC, 2 × RF, 900 °C) GPa.

**Figure 8:**
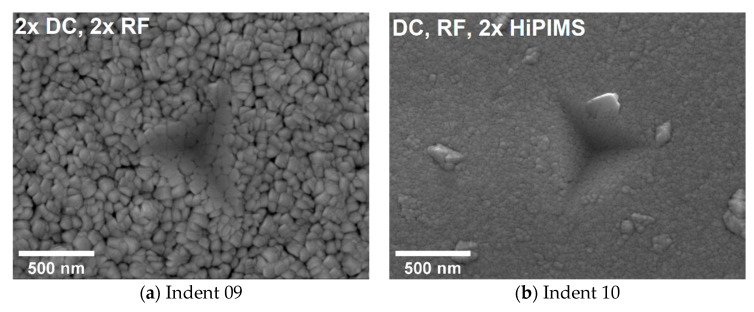

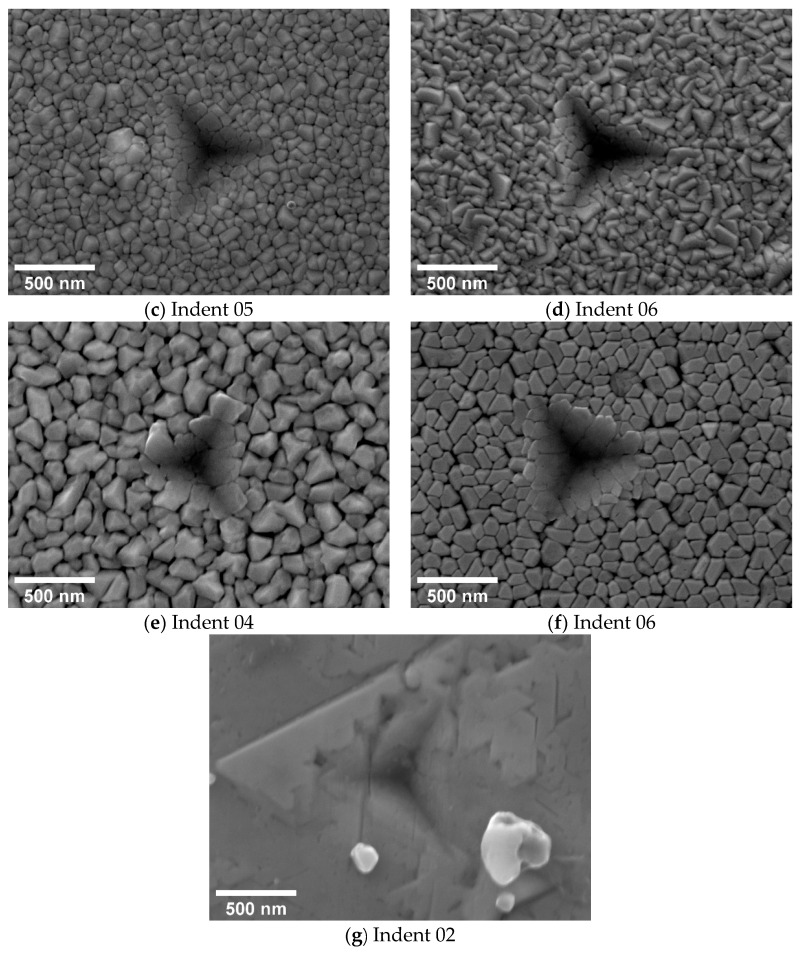
SEM images of representative nanoindentations out of the 10 made for each experimental condition, with the plasma generators used noted at the top of each column: (**a,b**) 25 °C, (**c,d**) 300 °C, (**e,f**) 500 °C, (**g**) 900 °C, 2 × DC and 2 × RF, where only three nanoindents could be located; none were found for 900 °C, 1 × DC 1 × RF 2 × HiPIMS.

The standard deviation varied over the deposition parameters as well. For deposition temperature of 300 °C, the standard deviations were particularly low. The standard deviation depended strongly on the surface quality and on surface morphology, such as roughness and especially the indenter hitting an anomalous feature in the films where these were prevalent. Note that since the nanoindentation was performed according to an automated grid pattern, each individual indentation point was not adjusted to avoid anomalous features. Therefore, after indentation, each of the 10 indents for each experimental condition were manually investigated by SEM. Figure 8 shows representative images of the indents. Load–displacement curves that significantly deviated from the average often correlated to full or partial indentation contact with a surface feature anomaly. For surfaces with strong morphologies, only a few or none of the indents could be found using SEM, namely, 900 °C, 2 × DC and 2 × RF, where only three nanoindents could be located, and 900 °C, 1 × DC 1 × RF 2 × HiPIMS, where none were found.

For samples with flatter surfaces and few or no anomalous features, such as those shown in Figure 8c,d, the scatter of nanoindentation results was the lowest. This shows that the deposition parameters can optimize thin films for mechanical characterization. For thin film nanoindentation, smooth surfaces are especially important as the indentation depth cannot be increased in order to minimize the surface effects. Here, the low final penetration depth of 100 nm is needed to avoid substrate influences. Therefore, to decrease the scatter of thin film nanoindentation results, optimizing the thin film surface becomes especially important. The relatively large scatter observed here was most likely largely due to surface morphology (roughness) [34,35], as indicated particularly for films deposited at 500 and 900 °C, although it is noted that the stress in thin films and the nanoscale grain size also play a role, along with pores [36,37] and cracks.

In an actual material library, which in our laboratory typically has a 342-point measurement grid on a Ø100 mm substrate, this manual investigation process of each indent is not feasible, nor is the time required for even the automated nanoindentation of 10 times at each area (leading to an approximately one week of measurement time). As the purpose of library screening is not to definitively characterize each unique composition but rather to start with a broad range of comparable materials and then use quick, automated instrumentation to identify composition regions with similar properties, trends in changes of properties and potentially compositions with sudden changes in functional property values. These much fewer, selected specific compositions would then be the subject of detailed, manual one-at-a-time investigation and, eventually, optimization. Although there were significant thin film effects and scatter, the results are still consistent with the nanoindentation hardness of 4.57 ± 0.29 GPa reported for microcrystalline CoCrFeNi wires [38].

Figure 7 and Figure 8 indicate that with a sufficient number of nanoindentation measurements, a representative average value can be obtained. However, the appropriate number of measurement repeats depends on the morphological homogeneity, both at the surface and in the thin film volume, and on the roughness. Measurement outliers might be excluded from the calculations if the indents can be located and examined at high magnification and when the material anomaly at cause is clear. It may be preferable to alter the growth conditions to a different structure zone region, where cracks, voids, anomalous growth features, and surface roughness are better suited to this measurement technique. In the case of CoCrFeNi studied here, the simplest and lowest temperature condition for this was a 300 °C deposition temperature using 2 × DC and 2 × RF generators. While equiatomic CoCrFeNi did not exhibit decomposition of the apparently single-phase solid solution at the temperatures and energies studied here (perhaps beginning at around 900 °C), at other elemental concentrations or with the addition of other elements, it may decompose at lower temperatures or be multiphase as deposited.

For automated, high-throughput measurements of combinatorial material libraries, there can be dozens or hundreds of composition locations being measured, with a fixed number of nanoindentation repetitions at each location. Minimizing the number of nanoindent repetitions at each composition, for example, to 3 or 5 indents, becomes important to reduce the time required to map the entire library. Automated examination of each indent is not currently possible, so optimizing the predominant morphologies in the library can contribute to less spread in limited-number measurement results. This in turn can enable detection of trends in property variation with composition or potentially with changes in phase(s).

## 4. Conclusions

Thin films of the multiple principal element alloy CoCrFeNi were deposited by magnetron sputtering, varying the deposition temperature and particle energies. Characteristic microstructures of the structure zone diagram were obtained in spite of the multielement complexity. The microstructures corresponding to zones 2 and 3 were found to shift to substantially lower homologous temperatures with increased particle energy input. For temperatures up to 500 °C and with the highest particle energy applied (1 × DC, 1 × RF, 2 × HiPIMS generators), CoCrFeNi remained in the fcc structure. At 900 °C the fcc texture changes, with indications of another phase forming, grain boundary grooving and recrystallization. Considerable variation in repeated nanoindentation measurement values were observed for some film morphologies, which could generally be attributed to indenter contact with anomalous growth features. These inhomogeneities can be reduced or eliminated by the choice of film temperature and particle energies to be in zone T or zone 1, which is 300 °C for CoCrFeNi, without the need for additional energy from HiPIMS generators. The result leads to automated, high-throughput nanoindentation mapping of combinatorial material libraries being more reproducible and sensitive for the detection of property trends and changes.

## Figures and Tables

**Table 1:** Overview of sputter conditions and resulting thin film compositions.

T	Power Supply				EDS Results ^1^			
(°C)	Cr	Fe	Co	Ni	Cr (at.%)	Fe (at.%)	Co (at.%)	Ni (at.%)
25	DC	DC	RF	RF	26	25	25	24
300					25	25	25	25
500					24	28	24	25
900					23	26	25	26
25	HiPIMS	DC	HiPIMS	RF	25	26	25	24
300					25	26	26	24
500					26	26	25	24
900					24	27	24	24

^1^ EDS values are the average of 10 adjacent measurements, with σ < 0.5 at.%. Because of rounding, values may not add up to 100%.

**Table 2:** Roughness (R_a_) determined in AFM and confocal laser scanning microscope (CLSM).

T	Power Supply				R_a_ (AFM)	R_a_ (CLSM)
(°C)	Cr (at.%)	Fe (at.%)	Co (at.%)	Ni (at.%)	(nm)	
25	DC	DC	RF	RF	8.0	7.0
300					3.5	2.0
500					8.0	5.0
900					8.8	12.0
25	HiPIMS	DC	HiPIMS	RF	3.0	2.0
300					3.3	2.0
500					4.0	4.0
900					21	33.0

**Table 3:** Total number of grains determined with surface image analysis.

T	Power Supply				Total Number of Grains
(°C)	Cr (at.%)	Fe (at.%)	Co (at.%)	Ni (at.%)	
25	DC	DC	RF	RF	3487
300					3140
500					1436
25	HiPIMS	DC	HiPIMS	RF	6200
300					3639
500					2439

**Table 4:** Columnar grain widths determined in TEM images.

T	Power Supply				Grain Size
(°C)	Cr (at.%)	Fe (at.%)	Co (at.%)	Ni (at.%)	(nm)
25	DC	DC	RF	RF	67
300					100
500					142
25	HiPIMS	DC	HiPIMS	RF	48
300					74
500					125

**Table 5:** Mean equivalent grain size calculated from the image-processed SEM micrographs.

T	Power Supply				Mean Equivalent Grain Size
(°C)	Cr (at.%)	Fe (at.%)	Co (at.%)	Ni (at.%)	(nm)
25	DC	DC	RF	RF	75
300					83
500					127
25	HiPIMS	DC	HiPIMS	RF	54
300					75
500					96
